# Aging modifies the immunological signature of pesticide exposure: increased circulating sTREM-1 and sTREM-2 in rural workers

**DOI:** 10.1007/s00420-026-02251-1

**Published:** 2026-11-10

**Authors:** José Rodrigues do Carmo Neto, Miller Caldas Barradas, Acácio Arlem Tomaz, Amanda de Oliveira Matos, Aline Arantes de Oliveira, Jefferson do Carmo Dietz, Amanda Beatriz Adriano da Silva, Daniel Francisco Xavier, Pedro Henrique dos Santos Dantas, Marcelle Silva-Sales, Daniela de Melo e Silva, Helioswilton Sales-Campos

**Affiliations:** 1 Laboratory of Mucosal Immunology and Immunoinformatics, Institute of Tropical Pathology and Public Health, Federal University of Goiás, Goiânia, 746050-050 Brazil; 2 Laboratory of Mutagenesis, Institute of Biological Sciences I, Federal University of Goiás, Goiânia, 74045-155 Brazil

**Keywords:** Pesticide exposure, Immune biomarkers, Immunotoxicity, Cytokines, TREM

## Abstract

Pesticides pose significant risks to human health through both environmental and occupational exposure. In agricultural contexts, occupational exposure is particularly prevalent and has been linked to the activation of inflammatory pathways and dysregulation of the immune response. Nevertheless, the acute and chronic impacts of pesticide exposure on biomarkers of innate immunity remain poorly characterized. In this context, the soluble forms of TREM-1 and TREM-2 have emerged as sensitive indicators of inflammation and/or been associated with neurodegenerative disorders, respectively, however, they have not yet been explored as occupational biomarkers of pesticide exposure. Thus, this study aimed to evaluate the influence of age, duration of exposure, and type of pesticide on circulating levels of sTREM-1 and sTREM-2 in a cross-sectional cohort of 168 rural workers from the state of Goiás, Brazil, stratified according to type of exposure and age (≤ 50 and ≥ 51 years). Serum levels of sTREM-1 and sTREM-2 were determined by enzyme-linked immunosorbent assays (ELISA). Sociodemographic and exposure-related variables were evaluated using parametric and nonparametric statistical tests, depending on the data distribution. Older individuals had significantly higher levels of sTREM-1 and sTREM-2, regardless of the type of pesticide they were exposed to. Age was correlated with increased biomarker concentrations, while the number of pesticides seemed not to influence these molecules. Longer exposure to pesticides was positively associated with higher levels of both biomarkers. Lifestyle factors (smoking and alcohol consumption) and history of previous pesticide poisoning modulated these effects in specific subgroups. These findings indicate that aging plays a central role in modulating sTREM-1 and sTREM-2 levels in pesticide-exposed populations, potentially modifying the immune effects associated with different types and combinations of compounds. In this context, TREM-related markers emerge as promising indicators of chronic immune activation in environmental and occupational settings.

**Supplementary Information:**

The online version contains supplementary material available at https://doi.org/10.1007/s00420-026-02251-1.

## Plain Language

Purpose: Pesticide exposure has been associated with inflammation and immune dysregulation, but its effects on biomarkers of innate immunity remain poorly characterized. This study aimed to investigate the associations of age, exposure duration, and pesticide type with circulating soluble triggering receptor expressed on myeloid cells 1 and 2 (sTREM-1 and sTREM-2) in agricultural workers.

Methods: This cross-sectional study included 168 rural workers from Goiás, Brazil, stratified by pesticide exposure type and age (≤50 and ≥51 years). Serum sTREM-1 and sTREM-2 levels were measured using enzyme-linked immunosorbent assays (ELISA). Sociodemographic and exposure-related variables were analyzed using parametric or nonparametric statistical tests, as appropriate.

Results: Older participants exhibited significantly higher sTREM-1 and sTREM-2 levels, regardless of pesticide exposure type. Age was positively associated with both biomarkers, whereas the number of pesticides used showed no apparent influence. Longer exposure duration was associated with increased levels of both biomarkers. Smoking, alcohol consumption, and previous pesticide poisoning also modulated these associations in specific subgroups.

Conclusion: Age appears to be an important factor associated with circulating sTREM-1 and sTREM-2 levels among pesticide-exposed rural workers. These findings suggest that cumulative exposure and individual characteristics may contribute to immune alterations in this population. TREM-related markers warrant further investigation as potential indicators of chronic immune activation in occupational settings.

**Supplementary Information:**

The online version contains supplementary material available at https://doi.org/10.1007/s00420-026-02251-1.

## Introduction

The use of pesticides in agriculture has grown significantly in recent decades worldwide, and Brazil is currently one of the world’s largest consumers of these compounds (De Araujo, 2023; Gaboardi, 2023; Souza, 2023). The impacts of this intensive use extend beyond the agricultural environment, reflecting in a widespread occupational and environmental exposure, especially in producing regions of Brazilian Midwestern, such as Mato Grosso and Goiás states, where pesticide use is intense, driven by the large-scale production of crops such as soybeans, corn, and sugarcane (De Campos, 2021; Massuquini, 2024). Studies indicate that most medium and large-sized farms regularly use herbicides, insecticides, and fungicides, although precise data on the proportion of pesticide-using farms or the average annual consumption per hectare are still limited (Lopes-Ferreira, 2022; Maino, 2022). However, there has been a continuous increase in the total volume of applied pesticides, particularly herbicides such as glyphosate, 2,4-D and atrazine, reflecting the expansion of agribusiness and the growth of monocultures (Brovini, 2021; Gonçalves, 2022).

Exposure to pesticides may occur through direct or indirect pathways. In the context of extensive agriculture, such as in Brazil, exposed populations encompass not only traditional agricultural workers but also individuals residing in rural settlements. In these areas, the boundaries between the work and living environments frequently overlap, leading to a complex dynamic of both occupational and continuous environmental exposure (Amazonas et al. 2024). Consequently, the broad term “rural workers” more accurately characterizes this diverse demographic. Direct exposure primarily affects rural workers during pesticide handling, preparation, and application. In contrast, indirect exposure occurs through environmental contamination of water, soil, food, and aerosols, as well as through domestic contact with pesticide residues, including contaminated work clothes, utensils, and household surfaces (Ahmad, 2024; Sarojmoni, 2022; Tudi et al., 2022). Epidemiological studies show that rural workers frequently report episodes of acute poisoning, reflecting the occupational vulnerability associated with the handling of these products. Furthermore, many biomarkers of pesticide exposure have been identified in populations residing near agricultural areas, indicating that environmental contamination can result in chronic exposure even among individuals who do not work directly with pesticides (De-Assis, 2021; Tudi et al., 2022).

Based on the direct immunotoxicity exerted by pesticides, components of the immune system, such as neutrophils, lymphocytes, and cytokines, are increasingly being discussed as potential biomarkers in pesticide exposure (Fierro-Barrientos et al. 2025). The pesticide exposure can induce alterations in cytokine production, loss of immunological tolerance, and the development of immunosuppressed states or hyper-reactive responses, although these phenomena are affected by the class of pesticide. For example, in adult Mexican flower farmers, IL-6 and IFN-γ serum levels were higher in those farmers exposed mainly to insecticides and fungicides (Godínez-Pérez et al. 2024). On the other hand, reduced serum levels of IL-2, IL-8, and IFN-γ were found in adult Italian farmers exposed to the pyrethroid insecticide cypermethrin (Costa et al. 2013). In another context, adult Italian workers exposed mainly to neonicotinoids, pyrethroids, and organophosphates showed higher levels of IL-22 (Fenga et al. 2014). Thus, based on the role of uncontrolled immune activation during exposure to pesticides, as well as the scarcity of immune biomarkers explored in this context, in addition to the observation of conflicting data in this regard, there’s a need for more studies seeking to understand the immunological mechanisms and inflammatory mediators that can be used as occupational biomarkers for pesticide exposure.

In recent years, the Triggering Receptor Expressed on Myeloid Cells (TREM) family of receptors has been explored in different infectious and non-infectious contexts. This family is composed of proteins belonging to the immunoglobulin superfamily, in which TREM-1 and TREM-2 are the most studied members (Bouchon et al. 2000; Colonna 2023). TREM-1 acts majorly as an amplifier of pro-inflammatory responses, in the production of pro-inflammatory cytokines and chemokines by myeloid cells, and also in the microbicidal functions of neutrophils (Bleharski et al. 2003; Prufer et al., 2014; Tammaro et al. 2017; Boufenzer et al. 2021; de Oliveira Matos et al., 2020). TREM-2, on the other hand, is a receptor with considerable plasticity, acting in the development, maturation and function of different cells, such as osteoclasts, microglia and dendritic cells, in the control of inflammation, but also in the promotion of phagocytosis and pro-inflammatory responses in some contexts (Hamerman et al. 2006; Turnbull et al. 2006; Kober and Brett 2017; Colmenares et al., 2024; Matos et al. 2024; Li et al. 2025; Xue et al. 2025). These receptors can also be found in their soluble form (named sTREM-1 and sTREM-2), which are formed through proteolytic cleavage by metalloproteinases or through alternative splicing (Gingras et al. 2002; Gomez-Pina et al. 2012), and are explored as biomarkers of several diseases.

Although few studies have investigated sTREM molecules in the context of pesticide exposure, there is a biological rationale for evaluating them. Chronic pesticide exposure is known to induce oxidative stress, low-grade inflammation, and neurotoxic effects. TREM-1 amplifies inflammatory responses in myeloid cells, whereas TREM-2 is involved in macrophage and microglial activation during tissue injury and neurodegeneration. In addition, changes in TREM signaling have been reported in other toxic environmental exposures, including cigarette smoke. Previous studies investigating pesticide exposure have predominantly focused on conventional inflammatory mediators, such as IL-6, TNF-α, IL-1β and IFN-γ, demonstrating that chronic exposure is capable of inducing systemic immune activation (Fierro-Barrientos et al. 2025; Souza et al. 2022). However, these cytokines primarily reflect transient inflammatory responses and may be influenced by several physiological and environmental conditions. In contrast, sTREM-1 and sTREM-2 are soluble receptors released by activated myeloid cells that provide complementary information on innate immune activation and immunoregulatory mechanisms (Zhou et al. 2025; Jolly et al. 2021a, b). While sTREM-1 amplifies inflammatory signaling, sTREM-2 has been associated with phagocytic activity, immune homeostasis and the resolution of inflammation (Zhang et al. 2025; Jolly et al. 2021a, b).

Therefore, investigating both receptors may provide additional insight into the long-term immunological consequences of occupational and environmental pesticide exposure beyond that offered by conventional inflammatory cytokines. Thus, considering the negative impact of these compounds towards different biological systems, including the immune system, and their ability to induce persistent inflammatory imbalances, investigating serum levels of sTREM-1 and sTREM-2 in individuals exposed directly or indirectly may help elucidate the underlying immune mechanisms and indicate the potential of these molecules as early markers of immunological alterations associated with occupational exposure.

## Materials and methods

### Study population and data collection

This cross-sectional study was conducted in a cohort of 168 rural workers from Goiás State, Central-West Brazil, including individuals with direct and indirect pesticide exposure. Participants were stratified according to age (≤ 50 and ≥ 51 years) and exposure type. Participants were not recruited from a pre existing clinical cohort or during medical appointments. They were identified through field recruitment in partnership with local health departments, city halls, and community leaders. These partnerships allowed our team to visit agricultural properties and rural settlements, where we explained the study objectives, the study procedures, and biomonitoring before inviting individuals to participate. The Free and Informed Consent Form (TCLE) was applied after participants agreed to take part in the study. The main objective was to compare serum levels of sTREM-1 and sTREM-2 between individuals directly or indirectly exposed to pesticides, also considering the effect of age (≤ 50 years and ≥ 51 years) as a possible modulating factor.

Individuals of both sexes, aged between 18 and 90 years, with a history of direct or indirect exposure to pesticides were included in the study. As excluding criteria the following aspects were used: (i) those who did not sign the Free and Informed Consent Form (FICF); (ii) individuals outside the established age range; and (iii) people with cognitive or physical limitations that could compromise their understanding and participation in the research procedures.

The individuals were categorized into four groups: directly exposed (rural workers exposed to pesticides who are occupationally involved in activities such as storing, mixing, loading, and spraying pesticides) aged 50 years or younger (*n* = 35) and aged 51 years or older (*n* = 67); individuals indirectly exposed to pesticides, defined as residents living near agricultural plantations and exposed through environmental pathways such as airborne pesticide dispersion and secondary domestic contact (e.g., washing contaminated work clothes, which allows them to accurately identify the specific pesticide formulations used by directly exposed family members), were categorized into two age groups: ≤50 years (*n* = 25) and ≥ 51 years (*n* = 41).The age cut-off of 50 years was used for stratification based on evidence of immunosenescence and inflammaging beginning in midlife. From this age onward, the innate immune system shows increased baseline inflammatory activity and reduced regulatory capacity, including within the myeloid compartment involved in TREM signaling. This stratification was applied to evaluate whether age modifies the inflammatory response associated with pesticide exposure.

All participants were informed in detail about the objectives and procedures of the study and were only included after signing the informed consent form. The protocol was approved by the Research Ethics Committee of the Federal University of Goiás (CAAE: 40325020.3.0000.8155) and conducted in accordance with Resolution No. 466/12 of the National Health Council, which regulates research involving human subjects in Brazil. Prior to biological sample collection, a questionnaire with open and closed questions was administered to all participants covering demographic characteristics (age and sex), lifestyle factors (smoking and alcohol consumption), anthropometric variables (height and weight), occupational history, and pesticide exposure characteristics. Exposure-related variables included exposure type (direct or indirect), years of pesticide exposure, history of acute pesticide intoxication, number of pesticide products used, specific pesticide products reported, and occupational activities involving pesticide handling (e.g., mixing, spraying, transportation, storage, disposal, and washing contaminated clothing), at a single baseline timepoint via face-to-face interviews by trained research personnel.

### Sample collection

Fifteen milliliters of peripheral blood were collected from each participant in vacuum tubes containing EDTA and clotting activator. The samples were transported at 4 °C to the Mutagenesis Laboratory (LabMut) at the Federal University of Goiás, where they were processed, frozen, and stored initially at − 20 °C and then at − 80 °C. They were then sent to the Mucosal Immunology and Immunoinformatics Laboratory (LIMIM) at the same institution, where sTREM-1 and sTREM-2 analyses were performed.

### sTREM-1 and sTREM-2 levels

The levels of sTREM-1 (Catalog DY1278B, Lot. P400973, R&D Systems, Minneapolis, USA) and sTREM-2 (Catalog DY1828-05, Lot. P394386, R&D Systems, Minneapolis, USA) were assessed in serum samples and quantified using an enzyme-linked immunosorbent assay (ELISA), according to the manufacturer’s instructions. Tetramethylbenzidine (TMB) (3,3,5,5-tetramethylbenzidine) was used as the substrate for the colorimetric reaction, and the optical density was measured with the Agilent BioTek 800 TS microplate reader (Agilent Technologies, Inc., Santa Clara, California, USA), using wavelengths of 450 nm and 570 nm. The result was expressed as pg/mL.

### Statistical analysis

Continuous variables were expressed as mean ± standard deviation (SD) or median with interquartile range (IQR), according to data distribution. These included age, serum sTREM-1 and sTREM-2 concentrations, years of pesticide exposure, and number of pesticides in correlation analyses. Categorical variables included sex, age group, exposure type, smoking, alcohol consumption, history of pesticide intoxication, BMI categories, and categorized exposure metrics. Statistical analyses were performed using GraphPad Prism 10.0 software (GraphPad Software, Boston, MA, USA) and RStudio: Integrated Development Environment for R. Posit Software, PBC, Boston, MA (2024). Shapiro–Wilk and Levene tests were performed to verify normality and homoscedasticity. The Student’s t-test was used for continuous variables, and the Fisher’s exact test was used for categorical variables.

For the immunological biomarkers sTREM-1 and sTREM-2, multivariate analyses were performed to assess the joint effects of age group and exposure type using Permutational Multivariate Analysis of Variance (PERMANOVA), implemented with the adonis2 function in the vegan package (Oksanen et al. 2024) in R (R Core Team 2024). In addition to age group and exposure type, the PERMANOVA model included body mass index category (BMI), smoking status, alcohol consumption, and self-reported history of pesticide intoxication as explanatory variables, allowing a comprehensive assessment of lifestyle and exposure-related factors on the combined biomarker profile. These variables were selected a priori because they are recognized factors that may influence systemic inflammatory responses and could potentially confound the relationship between pesticide exposure and circulating sTREM-1 and sTREM-2 levels. Analyses were based on Euclidean distance matrices calculated from log-transformed biomarker concentrations [log(x + 1)].

Homogeneity of multivariate dispersions was evaluated using PERMDISP, implemented with the betadisper function (Anderson 2006) in vegan, and assessed by permutation tests (9,999 permutations). Multivariate patterns were visualized using Principal Coordinates Analysis (PCoA). In addition, separate linear models were fitted for each biomarker to evaluate the effects of age group, exposure type, and their interaction. Unlike the multivariate PERMANOVA, these models were designed to estimate the independent and interactive effects of the primary study factors and therefore did not include additional covariates. Model assumptions were checked using residual diagnostics, and statistical significance was assessed by analysis of variance (ANOVA). A p-value < 0.05 was considered statistically significant.

## Results

### General characteristics of individuals indirectly or directly exposed to pesticides

**Fig. 1:**
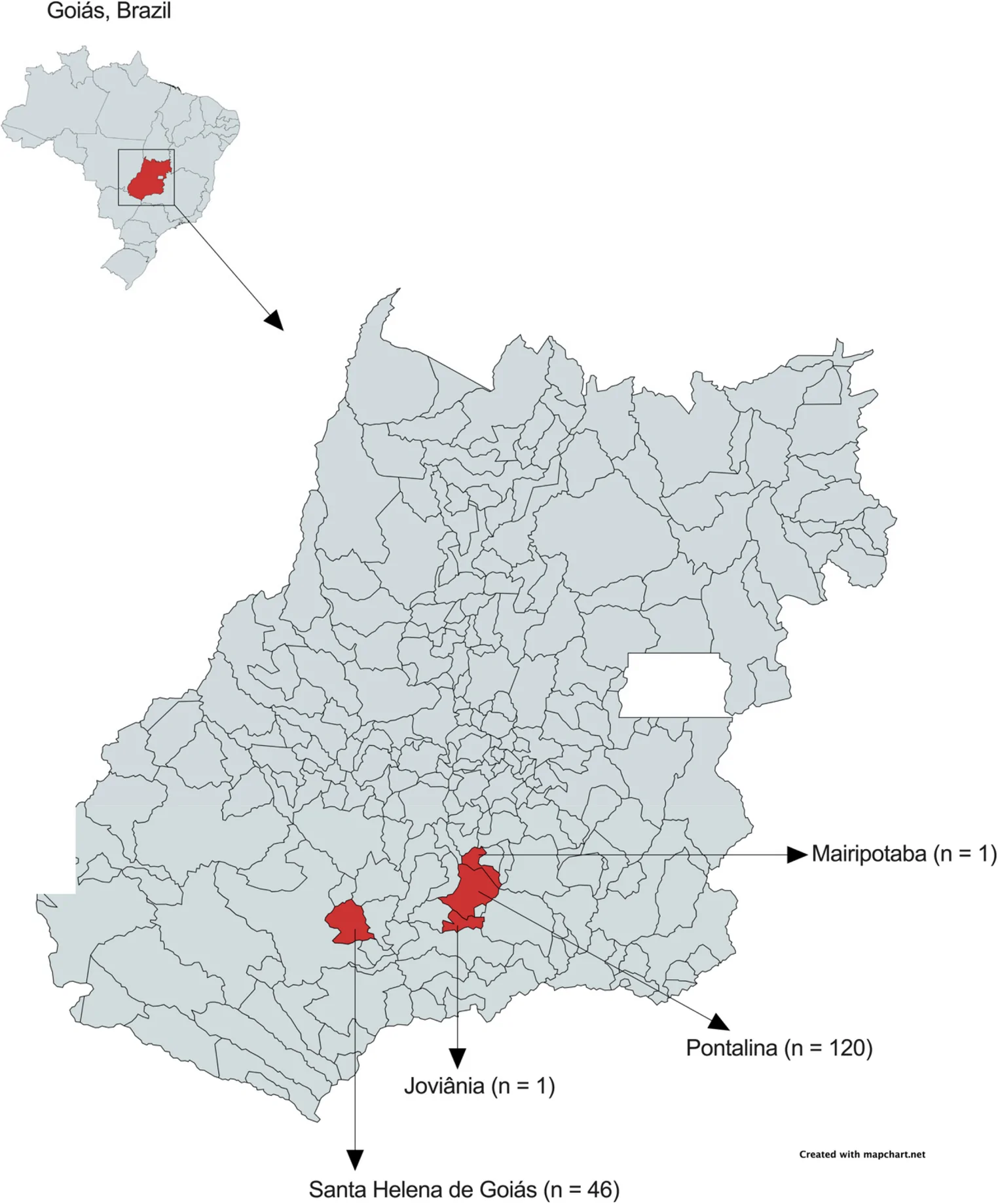
Geographic distribution of the study participants across municipalities in Southern and Southwestern Goiás, Brazil. Numbers indicate the sample size in each municipality: Pontalina (*n* = 120), Santa Helena de Goiás (*n* = 46), Joviânia (*n* = 1), and Mairipotaba (*n* = 1)

The study included 168 individuals working in the state of Goiás, Brazil (Fig. 1). Most participants, 71.4% (*n* = 120), were from the municipality of Pontalina, located in the Southern region of Goiás, followed by 27.4% (*n* = 46) from Santa Helena de Goiás, situated in the Southwestern region of the state. A smaller proportion of participants, totaling 1.2%, worked in the municipalities of Joviânia (*n* = 1) and Mairipotaba (*n* = 1), both also located in the Southern region of Goiás. Regarding exposure type, 60.7% of the individuals were directly exposed to pesticides, whereas 39.3% were indirectly exposed.

To assess the influence of age on serum sTREM-1 and sTREM-2 levels, participants were grouped into two age groups: ≤50 years and ≥ 51 years. Among those indirectly exposed, 37.8% were ≤ 50 years old and 62.2% were ≥ 51 years old. In the directly exposed group, 34.3% were ≤ 50 years old and 65.7% were ≥ 51 years old. Subsequent analyses were performed considering both the type of exposure and the age group simultaneously (Table 1).

**Table 1:** Sociodemographic characteristics of individuals indirectly or directly exposed to pesticides

	Exposure type		*p* value
Exposure^a^	Indirect	Direct	
	66	102	
Meand Age^b^	55.60 ± 14.55	55.66 ± 13.85	
Sex^a^			**< 0.0001**
Male	23	83	
Female	43	19	
BMI^a^			0.054
< 18.5	2	-	
18.5–24.9	18	40	
25–30	22	38	
> 30	24	23	
Smoke status^a^			0.4617
Yes	17	29	
No	36	46	
Former smoker	13	27	
Alcohol consumption^a^			0.2075
Yes	29	59	
No	23	29	
Previous alcohol consumption	14	14	
Acute Pesticide Poisoning^a^			**< 0.0001**
Yes	9	49	
No	57	53	
Pesticides number^a^			**< 0.0001**
1–7	12	77	
8–16	-	25	
Not informed	54	-	
Exposure time^a^			-
≤ 25 years	-	45	
≥ 26 years	-	57	

^a^ Absolute number

^b^ Mean age of the study population

### Relationship between sTREM-1 and sTREM-2 levels and the age of individuals and type of pesticides

**Fig. 2:**
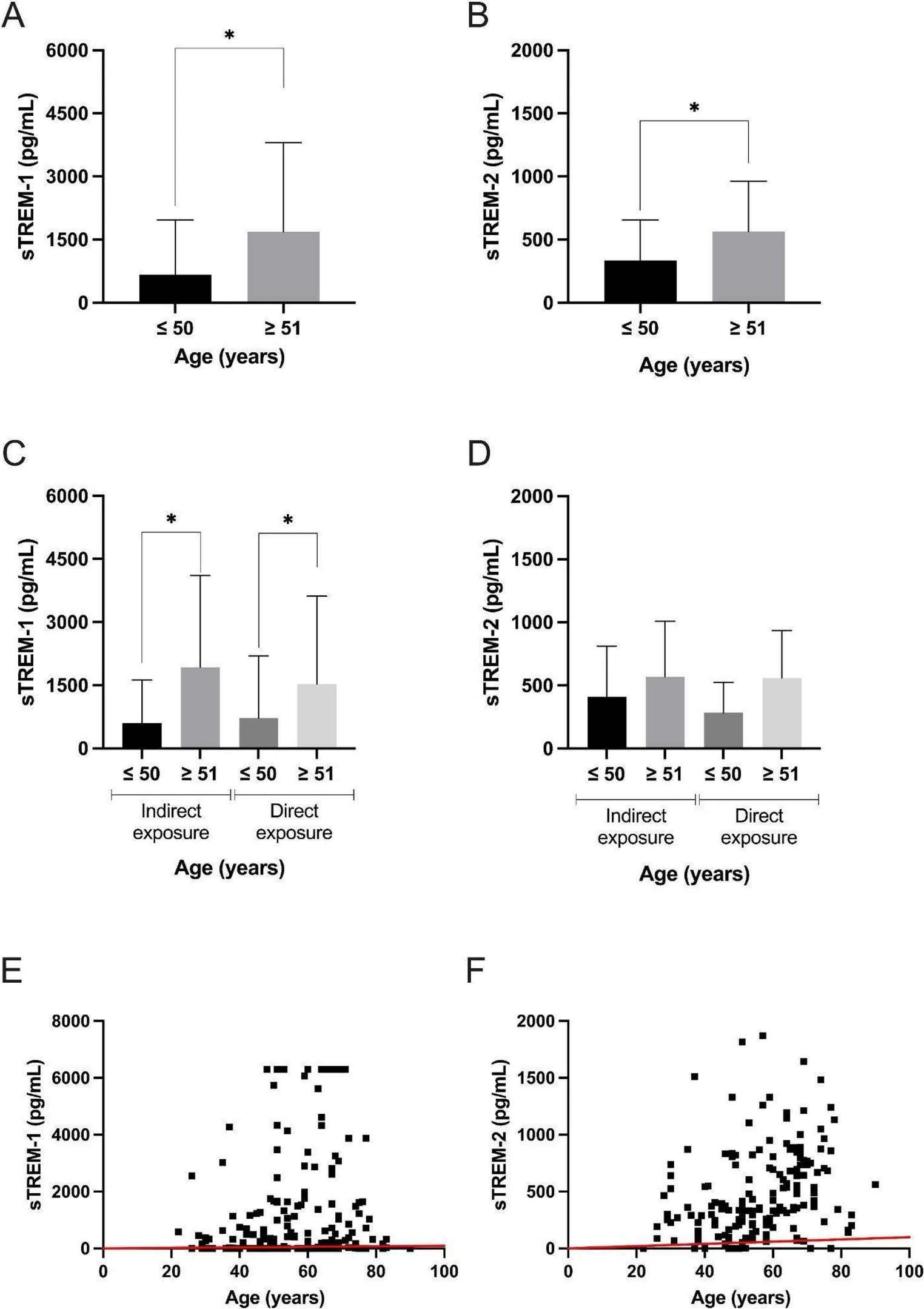
Levels of sTREM-1 and sTREM-2 in the serum of individuals indirectly and directly exposed to pesticides.**A** sTREM-1 levels in the serum of indirectly or directly exposed workers, grouped and divided into ≤ 50 years and ≥ 51 years of age. **B** sTREM-2 levels in the serum of indirectly or directly exposed workers, grouped and divided into ≤ 50 years and ≥ 51 years of age. **C** Serum sTREM-1 levels in indirectly or directly exposed workers, separated and divided into ≤ 50 years and ≥ 51 years of age. **D** Serum sTREM-2 levels in indirectly or directly exposed workers, separated and divided into ≤ 50 years and ≥ 51 years of age. **E** Correlation between serum sTREM-1 and age. **F** Correlation between serum sTREM-2 and age. Samples obtained from serum of individuals aged ≤ 50 years and aged ≥ 51 years, indirectly and directly exposed to pesticides. Data expressed in pg/mL. * statistical difference with *p* < 0.05. sTREM-1: Soluble form of Triggering receptor expressed on myeloid cells 1. sTREM-2: Soluble form of Triggering receptor expressed on myeloid cells 2

Initially, serum levels of sTREM-1 and sTREM-2 were assessed in all individuals, regardless of the type of pesticide exposure (Fig. 2A-B). Overall, individuals aged ≥ 51 years had significantly higher serum levels of sTREM-1 (Fig. 2A; *p* = 0.0005) and sTREM-2 (Fig. 2B; *p* < 0.0001) compared to those aged ≤ 50 years, regardless of the type of pesticide exposure.

To further examine these findings, participants were stratified by exposure type (indirect or direct), while maintaining the age categorization (Fig. 2C–D). For sTREM-1, the same pattern previously observed was maintained, with older age being associated with higher serum levels regardless of exposure type (Fig. 2C; indirect exposure, *p* = 0.0321; direct exposure, *p* = 0.0075). In contrast, no significant differences in sTREM-2 levels were detected between age groups when stratified by exposure type (Fig. 2D). Subsequently, correlation analyses were performed between age and circulating levels of soluble markers without stratification by exposure type (Fig. 2E–F). These analyses revealed a positive association between age and serum levels of both sTREM-1 (*p* = 0.0062, *r* = 0.2124) and sTREM-2 (*p* < 0.0001, *r* = 0.3569).

**Fig. 3:**
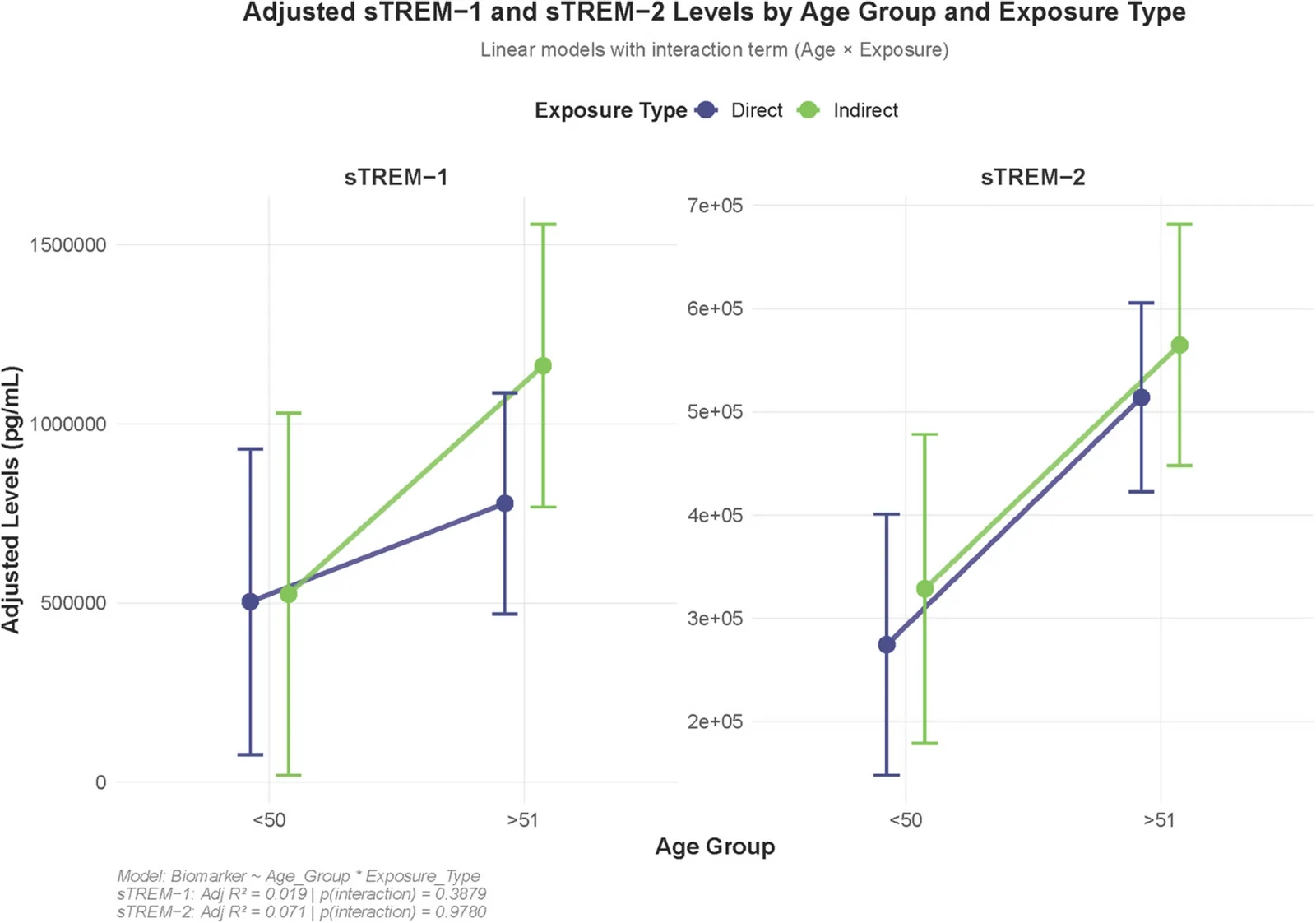
Adjusted mean circulating sTREM-1 and sTREM-2 levels (pg/mL) according to age group (< 50 and ≥ 51 years) and type of exposure (direct and indirect), estimated from a linear model including age group, exposure type, and their interaction. Points represent estimated marginal means, and vertical bars indicate 95% confidence intervals. The model shows a significant effect of age group on sTREM-2 levels, while neither exposure type nor the age–exposure interaction significantly influenced the biomarker concentrations

To determine whether age and exposure type independently influenced biomarker concentrations, a two-way ANOVA was conducted (Fig. 3). A significant main effect of age group was observed for sTREM-2 (F = 15.02, *p* < 0.001) and a modest effect was detected for sTREM-1 (F = 3.99, *p* = 0.047), with individuals aged ≥ 51 years presenting higher adjusted concentrations compared to those aged < 50 years. Consistent with the stratified and correlation analyses, no significant main effect of exposure type was detected for either biomarker, and the interaction between age group and exposure type was not statistically significant. Although statistically significant, age groups accounted for a relatively small proportion of the overall variability in biomarker levels (Adj R² ≤ 0.07).

To ensure a comprehensive evaluation, additional exploratory analyses were conducted to assess the impact of lifestyle factors (such as smoking, alcohol consumption, Body Mass Index) and previous history of acute pesticide poisoning on the circulating levels of sTREM-1 and sTREM-2. No statistically significant differences in these immunological markers were observed among the different lifestyle categories. The complete results of these exploratory analyses are available in the Supplementary Material (Supplementary Figure S1).

### Relationship between sTREM-1 and sTREM-2 levels and the quantity, years of exposure, and family of pesticides

**Fig. 4:**
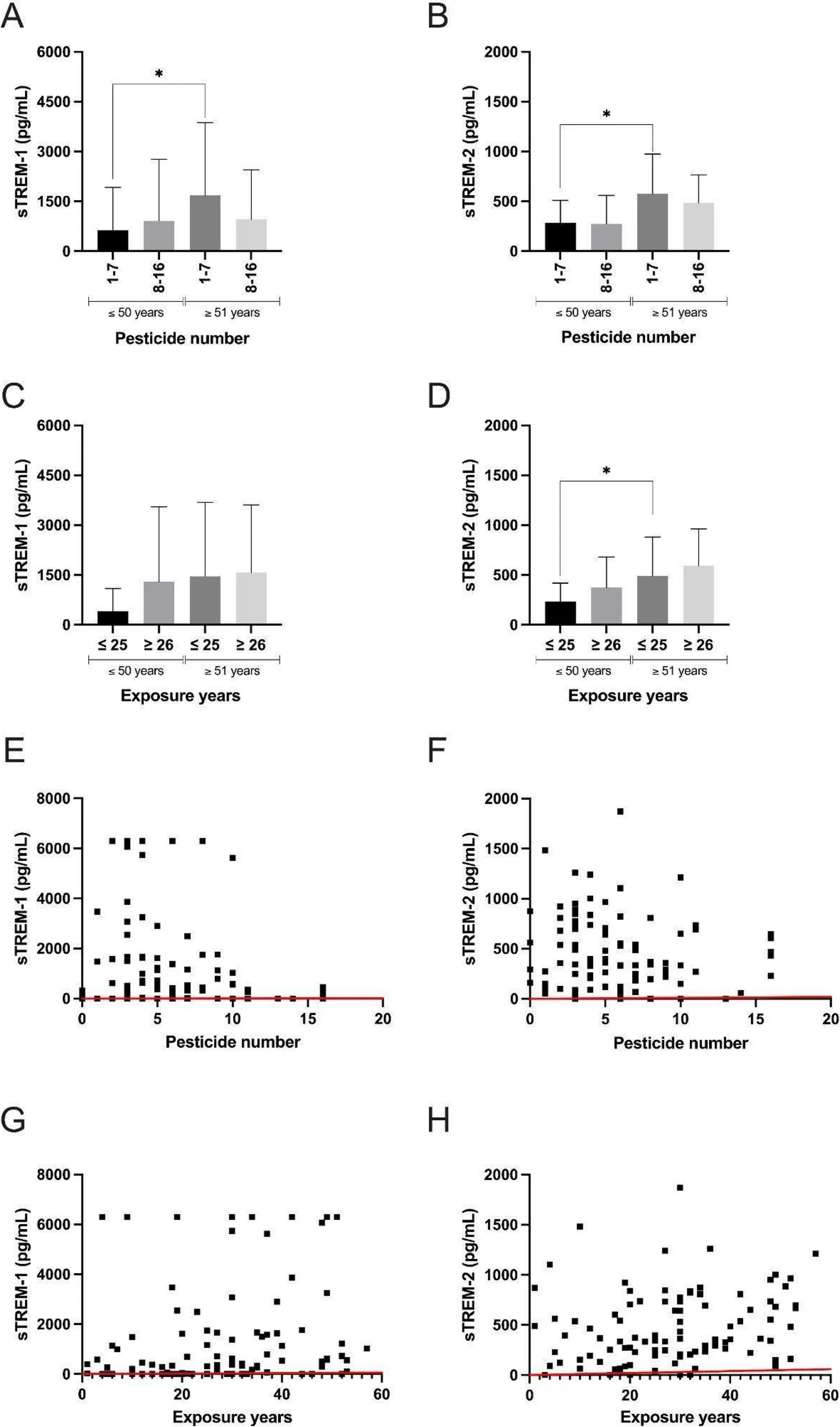
Relationship between sTREM-1 and sTREM-2 levels and factors such as number and years of exposure to pesticides. **A** sTREM-1 levels in the serum of indirectly or directly exposed workers, grouped and divided into those aged 50 years or younger and those aged 51 years or older. **B** sTREM-2 levels in the serum of indirectly or directly exposed workers, grouped and divided into those younger than or equal to 50 years of age and those older than or equal to 51 years of age. **C** sTREM-1 levels in the serum of indirectly or directly exposed workers, separated and divided into those aged 50 years or younger and those aged 51 years or older. **D** Serum sTREM-2 levels in workers exposed indirectly or directly, separated and divided into those aged 50 years or younger and those aged 51 years or older. **E** Correlation between serum sTREM-1 and number of pesticides. **F** Correlation between serum sTREM-2 and number of pesticides. **G** Correlation between serum sTREM-1 and years of exposure to pesticides. **H** Correlation between serum sTREM-2 and years of exposure to pesticides. Samples obtained from serum of individuals aged 50 years or younger and aged 51 years or older, indirectly and directly exposed to pesticides. Data expressed in pg/mL. * when statistically significant considering a p-value < 0.05

To assess the impact of the amount of pesticides, rural workers were categorized into two groups: 1 to 7 pesticides and 8 to 16 pesticides, maintaining the age as the main criteria for stratification (Fig. 4A-B). In this context, for both sTREM-1 (*p* = 0.0072) and sTREM-2 (*p* = 0.0014), age was a determining factor in the increase in markers among the groups exposed to 1 to 7 pesticides. On the other hand, this phenomenon was not observed between the age groups exposed to 8 or more pesticides.

To analyze whether exposure time could influence sTREM-1 and sTREM-2 levels, exposure was stratified into up to or equal to 25 years and greater than or equal to 26 years (Fig. 4C-D). In this case, no impact of exposure time on sTREM-1 levels was observed, regardless of the time of exposure. However, for sTREM-2, it was observed that age and time of exposure plays a role in the levels of this marker, especially in individuals exposed to pesticides for up to 25 years, when compared to individuals aged 50 years or younger (*p* = 0.0078).

Through correlation analysis, it was observed that the increase in sTREM-1 (Fig. 4E; *p* = 0.2713, *r* = -0.1111) and sTREM-2 (Fig. 4F; *p* = 0.2265, *r* = -0.1220) is inversely associated with the number of pesticides. Regarding exposure time, it was observed that the longer the exposure to pesticides, the higher the levels of sTREM-1 (Fig. 4G; *p* = 0.0088, *r* = 0.2608) and sTREM-2 (Fig. 54; *p* = 0.0081, *r* = 0.2635) levels, resulting in positive and significant correlations.

### Multivariate analysis of sTREM-1 and sTREM-2 profiles and the lifestyle and age of individuals exposed to pesticides

**Fig. 5:**
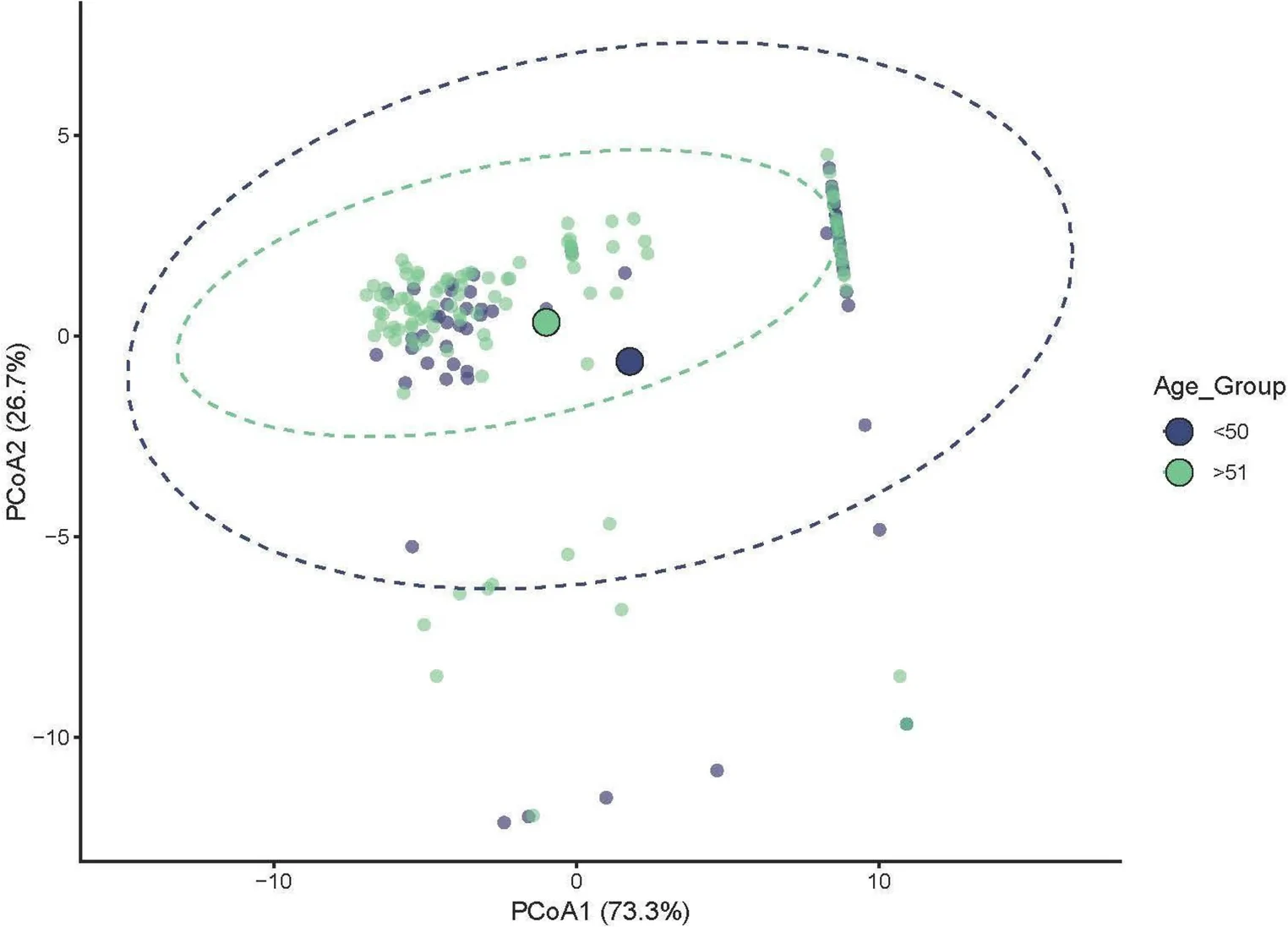
Principal Coordinates Analysis (PCoA) of circulating sTREM-1 and sTREM-2 stratified by age groups. Ordination was based on Euclidean distances calculated from scaled biomarker concentrations. Points represent individual participants, colored according to age group (< 50 years and ≥ 51 years). In addition to age group and exposure type, the PERMANOVA model included body mass index category (BMI), smoking status, alcohol consumption, and self-reported history of pesticide intoxication as explanatory variables, allowing a comprehensive assessment of lifestyle and exposure-related factors on the combined biomarker profile. Larger symbols indicate group centroids, and dashed ellipses represent within-group dispersion. Separation between age groups was mainly observed along the first PCoA axis, while the second axis reflected inter-individual variability

Multivariate analysis of the combined sTREM-1 and sTREM-2 data identified significant differences among the age and exposure groups (Fig. 5). A PERMANOVA detected a significant effect of age group and pesticide exposure on the multivariate biomarker profile (F = 3.64, R² = 0.062, *p* = 0.0015). indicating that these variables accounted for 6.2% of the observed multivariate variation.

Separation between age groups occurred primarily along the first PCoA axis, with displacement of group centroids. In contrast, the second PCoA axis showed substantial overlap among groups.

PERMDISP analysis also detected significant differences in multivariate dispersion among groups (F = 4.82, *p* = 0.003). Greater dispersion was observed among older individuals than among younger participants.

## Discussion

This study provides an integrated analysis of circulating sTREM-1 and sTREM-2 levels in individuals exposed to pesticides, demonstrating that age is the primary factor structuring the systemic inflammatory profile. In this regard, the type, intensity, and context of exposure, as well as lifestyle-related factors, are also important contributors. Across univariate, stratified, and multivariate analyses, advancing age appeared to be associated with higher sTREM-1 and sTREM-2 concentrations, independent of direct or indirect exposure. These findings position soluble TREM markers as sensitive indicators of age-associated immune activation in environmentally exposed populations, reinforcing the concept that chronic low-grade inflammation results from the interaction between biological aging and sustained environmental stressors rather than from isolated exposure events.

To understand the biomarker profile of the individuals included in this study, it is necessary to understand the profile of the population studied. Thus, with regard to demographic aspects, a discrepancy was observed between the sexes in terms of the type of exposure to pesticides, with men predominating in the direct exposure group, while women were concentrated in the indirect exposure group. This pattern reflects the typical occupational division in the agricultural environment, where men perform activities related to the preparation, handling, and application of pesticides, while women perform domestic and support functions, resulting in secondary/indirect exposures such as washing contaminated clothes, contact with application utensils, or proximity to spraying areas (Panis, 2022; Asmare et al., 2022; Deziel et al., 2015). This occupational differentiation not only highlights gender inequality in exposure conditions, but also suggests distinct mechanisms of contaminant absorption and accumulation between the sexes. This leads to divergent immune and inflammatory responses to the same toxic agents, as previously suggested by Asmare et al. (2022).

In fact, exposure to pesticides can induce different immune responses over time. Different studies report both the induction of a pro-inflammatory profile, with the presence of cytokines such as IFN-γ, TNF-α, and IL-6, and immunosuppression, especially in the plasma of exposed individuals (Lozano-Paniagua et al. 2024; Lopes-Ferreira et al. 2023). In this context, the present study investigated two new soluble markers recently associated with the activation of the immune response, sTREM-1 and sTREM-2, seeking to understand how their levels relate to age and type of exposure to pesticides.

sTREM-1 and sTREM-2 are products of proteolytic cleavage of their membrane-associated forms, an event that occurs after activation of their signaling pathways, resulting from the action of metalloproteinases (Colonna 2023; Gomez-Pina et al., 2007; Weiss et al. 2017). Thus, sTREM-1 and sTREM-2 have been used as markers of mTREM-1 and mTREM-2 activation (Jolly et al. 2021a, b) and, therefore, explored as diagnostic and/or prognostic biomarkers for conditions in which these receptors appear to be involved, such as infectious and non-infectious diseases. With regard to sTREM-1, its detection at high levels has already been reported in patients with sepsis (Chang et al. 2022), COVID-19 (de Nooijer et al. 2021), pneumonia (Gibot et al. 2004), rheumatoid arthritis (Choi et al. 2012), inflammatory bowel disease (Park et al. 2009), periodontitis (Bostanci et al. 2013), among others, with positive correlations between its concentration and poor prognosis, including death (Jedynak et al., 2018; Su et al. 2016; Geng et al. 2024; Van Singer et al. 2021).

The sTREM-2 has been investigated more intensively in neurological contexts and is considered a marker of microglial activation (Wunderlich et al. 2013; Schlepckow et al. 2023). Its detection in patients with Alzheimer’s disease, multiple sclerosis, and Parkinson’s disease (Weber et al. 2022; Warmenhoven et al.,^ 2024^; Wilson et al. 2020) has already been reported, and there is a direct association between its levels and lower cognitive decline and synaptic dysfunction (Morenas-Rodríguez et al. 2022; Ewers et al. 2019; Muñoz-García et al. 2025), although it has also been associated with worsening motor function (Zhang et al. 2023). In addition, TREM-2 has been implicated in dyslipidemia, atherosclerosis, and metabolic syndrome, with a direct correlation between serum sTREM-2 levels and the diagnosis of coronary heart disease and mortality from atherosclerosis (Liu et al. 2023; Zhu et al. 2024a, b). Thus, considering the broad inflammatory contexts in which sTREM-1 and sTREM-2 have been implicated/explored, the serum levels of these molecules were investigated in our study, which focused on individuals exposed to pesticides in different ways and at different intensities, given the growing evidence of the involvement of these substances in the immune system.

In the present study, aging was a determining factor for increased serum levels of sTREM-1 and sTREM-2, regardless of the form of exposure (direct/indirect). These results show that advancing age, combined with chronic environmental exposure, can potentiate systemic inflammatory processes and alter the regulation of myeloid pathways. It is important to highlight that the linear model analysis using adjusted marginal means (Fig. 3) demonstrated that age remained the only significant determinant of circulating sTREM-2 levels and, to a lesser extent, of sTREM-1 levels after adjustment, while neither exposure type nor the age–exposure interaction exerted a significant effect. This suggests that the age-related increase in sTREM-2 reflects intrinsic immune remodeling processes associated with aging, such as sustained activation of myeloid cells, altered phagocytic activity, and low-grade chronic inflammation, which may be exacerbated but not fundamentally dictated by the route or type of pesticide exposure (Bleve, 2022; Salminen 2019). Previous data indicate that prolonged exposure to pesticides is associated with sustained cytokine activation and oxidative stress, contributing to a persistent inflammatory state (Sule et al., 2022; Ledda et al., 2021; Costa et al., 2020). Thus, the increase in sTREM-1 and sTREM-2 levels observed in older individuals may reflect an adaptive response to low-grade inflammation induced by pesticides and aging. In addition, workers exposed directly or indirectly often report episodes of acute poisoning (Hailu et al., 2025; Coelho, 2024), and have detectable biomarkers of exposure even at environmental levels, reinforcing that continuous contact, even if indirect, is sufficient to modulate measurable immune responses in serum (Filippi et al., 2021; Sánchez-Alarcón et al, 2021). Notably, indirectly exposed individuals also had higher levels of sTREM-1 and sTREM-2, reinforcing that environmental and household contamination, common in rural areas, is sufficient to trigger measurable immune responses. This observation is consistent with Bravo et al. (2022), who reported that passive exposure to organophosphates and pyrethroids in family members of rural workers and populations living near croplands led to the expression of elevated biomarkers of inflammation and oxidative stress, even in the absence of direct occupational contact. The association between direct exposure and increased sTREM-1 corroborates the findings of Siskind et al. (2022), who demonstrated an elevation of this marker in contexts of persistent inflammation and exacerbated immune response to xenobiotic compounds.

Notably, the multivariate integration of sTREM-1 and sTREM-2 levels provides biological context for the univariate and stratified findings by demonstrating that age and pesticide exposure jointly shape the global innate immune activation profile. The significant PERMANOVA result indicates that these variables act in a coordinated manner to influence circulating sTREM levels, even though they explain a modest proportion of the total variance, which is expected in multifactorial human immune responses (Arsenault, 2024; Noh, 2024; Fan, 2023; Jolly, 2021a, 2021b). The ordination pattern observed in the PCoA suggests that aging promotes a gradual shift in the inflammatory landscape rather than discrete immune phenotypes, consistent with the concept of inflammaging and with the continuous age-related increases observed for both biomarkers (Baechle, 2023; Saavedra, 2023; Teissier, 2021). Moreover, the higher multivariate dispersion detected among older individuals indicates increased heterogeneity in immune regulation with advancing age as observed by Cervigel (2022), which may explain the context-dependent effects of lifestyle factors, pesticide poisoning, and exposure intensity observed in stratified analyses (Riemann, 2025). Collectively, these results suggest that pesticide exposure does not impose a uniform inflammatory signature but instead interacts with age-related immune remodeling, amplifying inter-individual variability and shaping distinct inflammatory trajectories within exposed populations (Fierro-Barrientos, 2025; Cestonaro, 2022; Ruiz-Arias, 2022).

Aging was also responsible for the increase in sTREM-1 and sTREM-2 when behavioral aspects were considered (smoking, alcoholism, and pesticidepoisoning), regardless of the type of exposure. However, considering the complexity of these phenomena, we chose to perform analyses categorizing the data according to the type of exposure. In this sense, for smoking, we observed that smoking induces an increase in sTREM-2 in indirectly exposed individuals. In fact, increased levels of TREM-2 and receptors involved in its activation pathway (DAP-12) have been demonstrated in the lungs of habitual smokers (Koth et al. 2010; Pintarelli et al. 2019). This increase has been linked to the induction of pulmonary inflammation and, consequently, to chronic lung diseases such as pulmonary emphysema (Koth et al. 2010) and lung cancer (Pintarelli et al. 2019). Interestingly, direct exposure to pesticides was able to reduce sTREM-2 levels when compared to indirect exposure in smokers aged ≥ 51 years. Although no study has demonstrated the relationship between sTREM-2, smoking, and pesticides, we believe that this phenomenon occurs due to immunosuppression caused by exposure to pesticides. As for alcoholism, no relationship was observed between this behavior and sTREM-1 and sTREM-2 levels, only maintaining the impact of age on both biomarkers.

To further understand the factors modulating these immune responses, we explored the influence of lifestyle and clinical variables (smoking, alcohol consumption, body mass index, and history of acute pesticide poisoning). While previous studies link smoking (Koth et al. 2010; Pintarelli et al. 2019), acute poisoning (Yen et al. 2021; Kim et al. 2022), and obesity (Schmidt et al. 2015) to immune alterations, our data revealed that age remained as the overriding determinant of increased sTREM-1 and sTREM-2 levels across these subgroups (Supplementary Figure S1). Although some specific variations were noted, such as a potential compensatory reduction of sTREM-2 in younger directly exposed individuals without poisoning, these behavioral and clinical factors did not drastically override the combined effects of aging and chronic environmental exposure. This reinforces our primary finding that the observed myeloid dysregulation is driven fundamentally by the continuous interplay between immunosenescence and sustained pesticide exposure, rather than by isolated lifestyle variables or acute toxic events.

Regarding the amount and duration of exposure, it was observed that the number of pesticides was not a determining factor in the increase in sTREM-1 and sTREM-2 levels. However, the length of exposure showed a positive correlation with the increase in the aforementioned biomarkers. This suggests that the cumulative effect of chronic exposure, rather than the diversity of compounds, is the main factor associated with the activation of myeloid inflammatory pathways. This was previously demonstrated by Liu et al. (2021) in a study conducted with 1,723 participants in the National Health and Nutrition Examination Survey (NHANES, 2011–2012). This pattern was also described by Purwandhono et al. (2024) in farmers exposed to organophosphates for long periods, in whom exposure time was a better predictor of immune dysfunction than single-dose exposure.

Despite the observation that sTREM-1 and sTREM-2 levels can be used as occupational biomarkers in individuals exposed to pesticides, our study presents some limitations. Among them, the absence of an age-matched control group with little or no environmental exposure to pesticides may limit further interpretation of our data. The heterogeneity of exposure times and types of pesticides may also impact the analysis, in addition to not considering comorbidities for comparison between the groups included. Nevertheless, the findings of this study suggest that age may be an important factor associated with increased levels of these biomarkers in rural workers. Thus, it reinforces the importance of analyzing the impact of sTREM-1 and sTREM-2 levels on the immune system and, consequently, on pesticide-mediated toxicity in chronically exposed populations.

## Supplementary Information

Below is the link to the electronic supplementary material.

Supplementary Material 1.
