# Supplementary material for: Aging modifies the immunological signature of pesticide exposure: increased circulating sTREM-1 and sTREM-2 in rural workers
Source: Int Arch Occup Environ Health. 2026 Nov 10;99(9):62. doi: 10.1007/s00420-026-02251-1 (PMC13751714; doi:10.1007/s00420-026-02251-1)
Supplement: Supplementary file 1 [file 420_2026_2251_MOESM1_ESM.docx]

**Supplementary Material**

**Supplementary Figure S1. Relationship between sTREM-1 and sTREM-2 levels and factors such as smoking, alcoholism, acute pesticide poisoning, and BMI in the serum of individuals indirectly and directly exposed to pesticides.**

(A) Relationship between smoking and sTREM-1 levels in the serum of workers, grouped according to type of exposure (indirect + direct).

(B) Relationship between smoking and sTREM-2 levels in the serum of workers, grouped according to type of exposure (indirect + direct).

(C) Relationship between smoking and sTREM-1 levels in the serum of workers, individualized, according to type of exposure (indirect or direct).

(D) Relationship between smoking and sTREM-2 levels in the serum of workers, individualized, according to type of exposure (indirect or direct).

(E) Relationship between alcoholism and sTREM-1 levels in the serum of workers, grouped according to type of exposure (indirect + direct).

(F) Relationship between alcohol consumption and sTREM-2 levels in the serum of workers, grouped according to type of exposure (indirect + direct).

(G) Relationship between alcohol consumption and sTREM-1 levels in the serum of workers, individualized according to type of exposure (indirect or direct).

(H) Relationship between alcoholism and sTREM-2 levels in the serum of workers, individualized according to the type of exposure (indirect or direct).

(I) Relationship between poisoning and sTREM-1 levels in the serum of workers, grouped according to the type of exposure (indirect + direct).

(J) Relationship between poisoning and sTREM-2 levels in the serum of workers, grouped according to type of exposure (indirect + direct).

(K) Relationship between poisoning and sTREM-1 levels in the serum of workers, individualized according to type of exposure (indirect or direct).

(L) Relationship between poisoning and sTREM-2 levels in the serum of workers, individualized, according to type of exposure (indirect or direct).

(M) Relationship between BMI and sTREM-1 levels in the serum of workers, grouped according to type of exposure (indirect + direct).

(N) Relationship between BMI and sTREM-2 levels in the serum of workers, grouped according to type of exposure (indirect + direct).

Samples obtained from serum of individuals aged 50 years or younger and 51 years or older, indirectly and directly exposed to pesticides. Data expressed in pg/mL. * when statistically significant considering p < 0.05. sTREM-1: Soluble form of Triggering receptor expressed on myeloid cells 1. sTREM-2: Soluble form of Triggering receptor expressed on myeloid cells 2.
